# Is It Worth Considering Multicentric High-Grade Glioma a Surgical Disease? Analysis of Our Clinical Experience and Literature Review

**DOI:** 10.3390/tomography7040045

**Published:** 2021-10-05

**Authors:** Francesco Guerrini, Lucio Aniello Mazzeo, Giorgio Rossi, Mariarosaria Verlotta, Mattia Del Maestro, Angela Dele Rampini, Alessandro Pesce, Marco Viganò, Sabino Luzzi, Renato Juan Galzio, Andrea Salmaggi, Giannantonio Spena

**Affiliations:** 1 Unit of Neurosurgery, Department of Neurosciences, Hospital A. Manzoni, 23900 Lecco, Italy; frague21@gmail.com (F.G.); la.mazzeo@asst-lecco.it (L.A.M.); m.verlotta@asst-lecco.it (M.V.); giannantonios@gmail.com (G.S.); 2 Unit of Neurosurgery, Department of Clinical, Surgical, Diagnostic and Pediatric Sciences, University of Pavia, 27100 Pavia, Italy; mattiadelmaestro@gmail.com (M.D.M.); angeladele.rampini@gmail.com (A.D.R.); sabino.luzzi@unipv.it (S.L.); renato.galzio@unipv.it (R.J.G.); 3 Unit of Neurosurgery, Hospital S. M. Goretti, 04100 Latina, Italy; 4 Unit of Pathology, Department of Oncology, Hospital A. Manzoni, 23900 Lecco, Italy; gi.rossi@asst-lecco.it; 5 Orthopedic Biotechnology Lab, IRCCS Orthopedic Institute Galeazzi, 20161 Milan, Italy; m.viga85@gmail.com; 6 Unit of Neurology, Department of Neurosciences, Hospital A. Manzoni, 23900 Lecco, Italy; a.salmaggi@asst-lecco.it

**Keywords:** glioblastoma, MRI, multifocal, brain, tumor

## Abstract

Introduction. The simultaneous presence of multiple foci of high-grade glioma is a rare condition with a poor prognosis. By definition, if an anatomical connection through white matter bundles cannot be hypothesized, multiple lesions are defined as multicentric glioma (MC); on the other hand, when this connection exists, it is better defined as multifocal glioma (MF). Whether surgery can be advantageous for these patients has not been established yet. The aim of our study was to critically review our experience and to compare it to the existing literature. Materials and Methods. Retrospective analysis of patients operated on for MC HGG in two Italian institutions was performed. Distinction between MC and MF was achieved through revision of MR FLAIR images. Clinical and radiological preoperative and postoperative data were analyzed through chart revision and phone interviews. The same data were extracted from literature review. Univariate and multivariate analyses were conducted for the literature review only, and the null hypothesis was rejected for a *p*-value ≥ 0.05. Results. Sixteen patients met the inclusion criteria; male predominance and an average age of 66.5 years were detected. Sensory/motor deficit was the main onset symptom both in clinical study and literature review. A tendency to operate on the largest symptomatic lesion was reported and GTR was reached in the majority of cases. GBM was the histological diagnosis in most part of the patients. OS was 8.7 months in our series compared to 7.5 months from the literature review. Age ≤ 70 years, a postoperative KPS ≥ 70, a GTR/STR, a second surgery and adjuvant treatment were shown to be significantly associated with a better prognosis. Pathological examination revealed that MC HGG did not originate by LGG. Conclusions. MC gliomas are rare conditions with high malignancy and a poor prognosis. A maximal safe resection should be attempted whenever possible, especially in younger patients with life-threatening large mass.

## 1. Introduction

Glioblastoma Multiforme (GBM) represents the most common primary brain tumor, with an incidence of 2–3 cases/100,000 people with males being more affected than females [1]. Considering the 2016 World Health Organization Tumor Classification, the mutation of IDH1/2 genes was introduced as an important prognostic factor, where IDH mutated GBM shows a longer history and a better therapeutic response that confers a greater overall survival compared to the IDH wild type GBM [2,3,4]. 

Although multiple neoplastic brain lesions are typical of secondary metastatic disease, the simultaneous presence of two or more lesions has been described in around 2% to 9% of all gliomas [5]. If it occurs, distinction should be made regarding the multifocal (MF) or multicentric (MC) origin of the tumor.

Considering the Batzdorf and Malamud criteria, a MF glioma is a condition where a diffusion along white matter association tracts can be hypothesized. In particular, neoplastic cells can migrate through natural commissural route (i.e., corpus callosum) or they can exploit normal cerebrospinal fluid circulation. On the contrary, if the origin of multiple lesions in different lobes or/and hemispheres cannot be ascribed to predictable routes, a MC glioma is more likely [5]. This latter is a rare entity with a worse prognosis compared to unifocal HGG and a clear therapeutic strategy has not been established yet. However, in some selected cases, where one lesion presents a larger volume or is clearly symptomatic, although not life threatening, surgical resection can be indicated in order to reduce the mass effect and establish a correct histological and molecular diagnosis. 

Here we present an analysis of our experience in the treatment of MC HGG, along with an up-to-date systematic review of the literature, trying to shed light on the role and usefulness of surgery in these rare and particularly malign entities.

## 2. Materials and Methods

### 2.1. Clinical Study

A retrospective study of patients treated for MC glioma at two Neurosurgical Departments (A. Manzoni Hospital, Lecco, Italy and San Matteo Hospital, Pavia, Italy) between 2015 and 2018 were considered eligible for the study. From a series of 320 HGG, those patients matching a diagnosis of multicenter or multifocal HGG were studied. All MRI were reviewed in order to distinuish between MC and MF. To do so, FLAIR T2-weighted MRI sequences were evaluated and in case a diffusion pathway was found between one or more lesions, the case was then excluded and classified as a MF glioma. Other exclusion criteria were: diagnosis of LGG and a previous intervention for an unifocal glioma; patients with clinical, radiological and pathological incomplete data or with metacronous lesions.

For every patient we considered: sex, age, clinical onset and preoperative Karnofsky Performance Status (preKPS); a supra- or infratentorial localization; ependymal involvement, defined by at least one lesion close to the ventricle. The extent of resection (referred to the single lesion resected) was evaluated on postoperative T1-weighted MRI sequences with gadolinium, considering a GTR a removal ≥ 90% and an STR an asportation < 90% [6,7]. Pathological data included mutations of IDH1/2, ATRX and TP53, proliferative index (Ki67), EGFR overexpression and 1p19q codelection; WHO 2016 classification system was applied. Postoperative clinical condition and adjuvant treatment were analyzed, considering the occurrence of a second resection. Finally, overall survival (OS) was calculated in months.

### 2.2. Review of Literature

Review of literature was effectuated through PubMed/EMBASE using the keywords “glioma”, “glioblastoma”, “multicentric” and “multifocal”. Studies without detailed or complete data for every patient were excluded. Moreover, cases with metachronous, low-grade lesions and spinal cord involvement were not included. The presence of a familiar syndrome constituted another exclusion criteria. We only considered English-language papers. Whenever it was possible, clinical study corresponding data were extracted for every patient described into the review included papers. 

### 2.3. Statistical Analysis

Concerning the clinical study, the analysis was limited to absolute and relative frequencies of categorical variables as well as descriptive statistics related to demographic and clinical data.

Statistical analysis was performed on patients’ data retrieved from literature using the open-source software RStudio (RStudio, Boston, MA, USA, 2019). Univariate and multivariate analysis were conducted. Significant OS-associated parameters were obtained applying log-rank test and Cox regression models. Null hypothesis was rejected for *p* value ≤ 0.05. Null hypothesis was rejected for *p* value ≤ 0.05.

## 3. Results

### 3.1. Clinical Study

From a database of 320 HGG, 24 were defined as MC and among these 16 patients were operated on and matched the inclusion criteria. There were 11 male (68.8%) and 5 female (31.2%) with a mean age of 66.5 years. Clinical onset consisted of motor and/or sensitive deficit in seven cases (43.8%), a language and/or cognitive disturbance in eight patients (50%) and only headache in two cases (12.5%); partial or generalized seizures were registered in 5 cases (31.8%). Preoperative mean KPS was 87.

The lesions had a purely supratentorial localization in 14 patients (87.5%) and an ependymal involvement was registered in seven cases (43.8%). Considering surgical data, a GTR was reached in 10 patients (62.5%), while in five patients we performed a biopsy (31.3%).

Pathology revealed a GBM in 14 patients (87.5%); in the remaining cases, an anaplastic oligodendroglioma and an anaplastic astrocytoma were diagnosed. All samples were IDH wild-type while ATRX resulted mutated in three cases; EGFR amplification was diagnosed in all but one cases. Ki67 proliferative index ranged between 10% and 70%. TP53 resulted mutated in all but two patients with values between 5 and 70%.

Postoperative KPS mean value was 79.4. This value tended to decrease at 65.3 and 45.7 at 3 months and 6 months follow-up respectively.

STUPP protocol adjuvant therapy was performed in nine patients (56.3%). Radiotherapy alone was performed in two cases. Chemotherapy was not performed in six patients. Overall survival was 8.7 months, with a range between 1 and 24 months (Table 1, Table 2 and Table 3).

### 3.2. Systematic Review of Literature

Thirty-three articles resulted from the research. After the application of exclusion criteria, 19 studies were excluded from the final analysis that was conducted on 14 papers published between 1990 and 2018. Fifty-five patients were analyzed. Male sex was predominant with 33 cases. Mean age was 58.7 years. At the clinical onset, a sensory and/or motor deficit was the most common symptom (44.2%), followed by headache (34.6%). Only six patients presented to observation with seizures. Preoperative mean KPS was 67.1.

Disease extension was reported in 54 cases and a purely supratentorial diffusion was shown in 42 cases (77.8%). Considering surgical data, a GTR was reached in 26 cases (55.6%) and a STR in seven patients (15.6%). Pathology confirmed clinical data, with a GBM diagnosed in 78.2% of samples. Combined adjuvant treatment was performed in 51.1% of patients; 12 cases were subdued to chemotherapy, only. Mean postoperative KPS, available for 20 patients only, was 73.5. Finally, OS was 7.5 months.

### 3.3. Statistical Analysis

**Figure 1:**
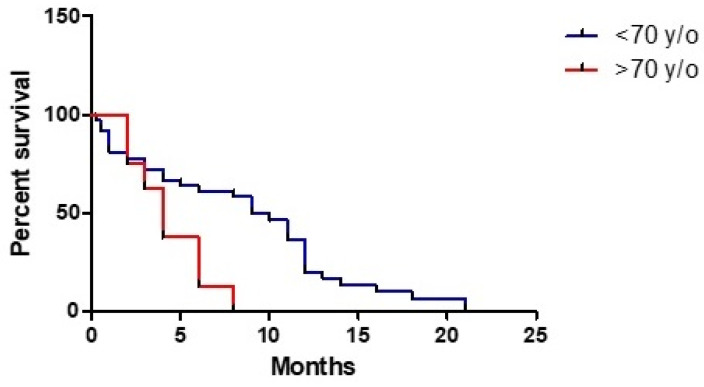
Prognostic role of age on overall survival.

Analysis of correlation between age and OS revealed a 4% higher relative risk for every year (HR 1.039, 95% CI: 1.010–1.069, *p* < 0.009) with a worse prognosis among patients with an age ≥70 years (HR 3.064, 95% CI: 1.271–7.385, *p* < 0.013) (Figure 1). On the contrary, sex was not shown as a prognosis factor (HR 0.819, 95% CI: 0.4204–1.595, *p* = 0.557) as well as preoperative KPS (HR 1.009, 95% CI: 0.987–1.031, *p* = 0.418). 

A purely supratentorial localization seemed to have a good tendency to play a protective role (HR 1.884, 95% CI: 0.938–3.785, *p* = 0.075).

**Figure 2:**
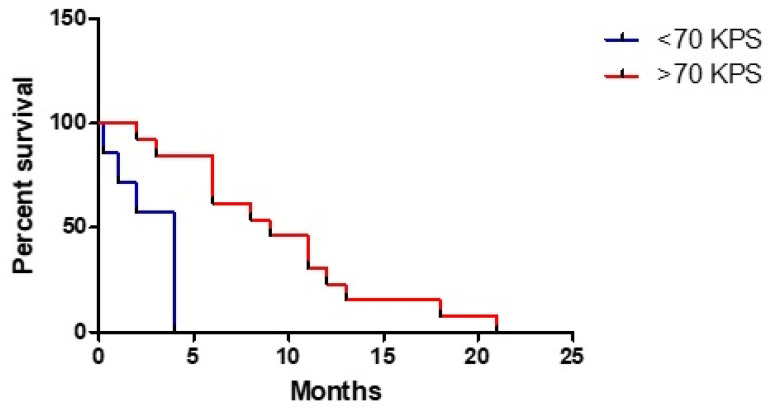
Role of postoperative KPS on overall survival.

Postoperative KPS was shown to have a good significative correlation with OS, in particular if it has a ≥ 70 value (HR 0.111, 95% CI: 0.023–0.536, *p* < 0.01) (Figure 2); considering a deeper analysis, for every KPS 10 points improvement a gain of 1.35 months emerged (HR 0.135, 95% CI: 0.938–0.994, *p* < 0.02). 

**Figure 3:**
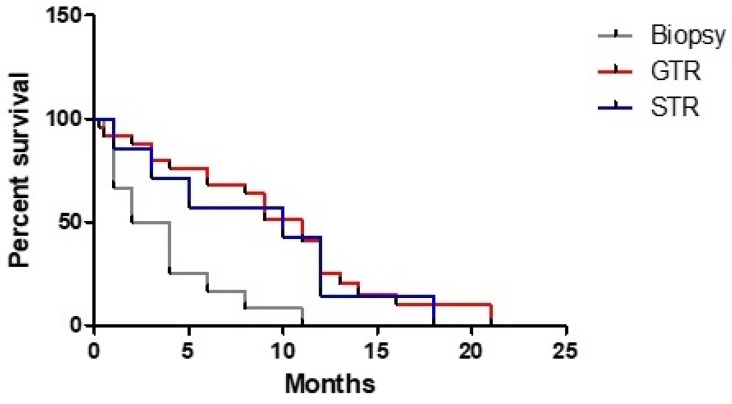
Correlation of extent of resection and overall survival.

A GTR (HR 0.258, 95% CI: 0.117–0.571, *p* < 0.001) and a STR (HR 0.308, 95% CI: 0.112–0.842, *p* < 0.022) appeared to have a strong role in determining a better prognosis if they were compared to the biopsy (Figure 3). 

Combined adjuvant chemo-radiotherapy was shown to exert a significant protective role (HR 0.417, 95% CI: 0.218–0.799, *p* < 0.009); moreover, if chemotherapy appeared to be significantly related to a higher OS (HR 0.192, 95% CI: 0.084–0.441, *p* = 0.0001), we cannot say the same thing for radiotherapy (HR 1.126, 95% CI: 0.276–4.591, *p* = 0.869). Nevertheless, advantages of adjuvant treatment were not confirmed for patients with a postoperative ≥ 70 KPS.

**Figure 4:**
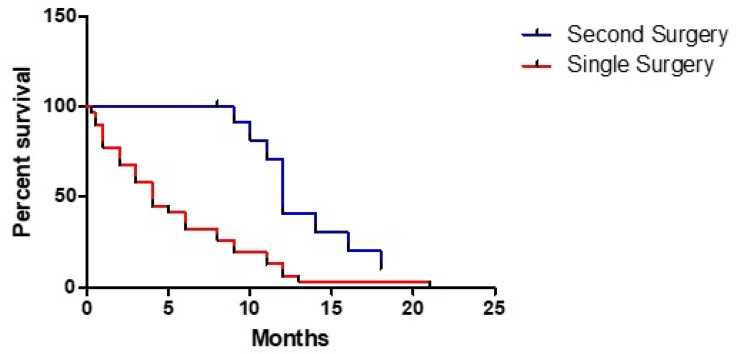
Different effect of a single or a second surgery on overall survival.

Finally, a second resection was related to a better prognosis (HR 0.266, 95% CI: 0.123–0.572, *p* < 0.001) (Figure 4).

## 4. Discussion

Glioblastoma is the most frequent primary brain tumor, and it usually presents as a unifocal lesion. However, multiple foci are reported with a percentage around 0.5–20% [8]. This last condition comprehends MF and MC gliomas.

As mentioned, multifocality is a condition for whom a microscopic diffusion pathway can be hypothesized. An interesting piece of information emerged from the study by Showalter et al.: no case of distant progression was observed in absence of local progression, showing that an evolutive pattern was the dominant one. In fact, considering adjuvant radiotherapy, no difference in terms of progression pattern, time to progression and overall survival was observed between a conformational and a whole brain technique [9].

On the contrary, our study concentrated on multicentricity, a rarer condition. In 1993, Kiritsis et al. stated that a distinction between MF and MC has no real clinical significance, suggesting the possibility of talking about an early MF glioma with a better prognosis and a late form with shorter survival [10]. This difference in terms of overall survival was confirmed by Hassaneen et al. (14.5 vs. 9.2 months) [11]. Nevertheless, multicentricity and multifocality seem to have a different physiopathology. In fact, according to the few studies analyzing molecular patterns of these entities, the former appeared to have an IDHwt tumorigenesis. This last data would suggest the absence of a low-grade progenitor and, as a consequence, an evolutive pattern would not seem probable [12]. Anyway, Sridharan et al. described two cases of LGG in the same patient without an apparent pattern of diffusion, making fall this condition into the concept of multicentricity [13].

Given the rarity of these entities and, consequently, the absence of uniform therapeutic strategies, we tried to analyze our clinical experience along with data available from the literature.

Patients included in our analysis ranged between 44 and 83 years of age, with a mean of 66.5 years. On the contrary, literature review showed a mean value of 58.7 years, close to that reported in Wang et al. [14]. Anyway, it seems that this pathology tends to involve the VI and VII decades of life, as for IDHwt glioblastoma, with a greater percentage of male sex gender both in clinical study (M/F 2.2) and literature review (M/F 1.6) [15,16]. Considering our experience, males would seem to have a better prognosis than females. On the contrary, statistical analysis did not reveal a difference in terms of OS between the two genders as seen in other studies. This apparent discrepancy could be explained with the fact that, considering the small size of the sample, the three female patients with a very short OS impacted more.

Considering clinical onset, our experience showed as language disturbance was the most common one, while it covered only 17.3% of literature cases; this difference could be explained by the fact that Salvati et al. did not analyze this type of symptom [17]. Anyway, Hassaneen et al. reported an incidence of 44% as for as multicentric subclass was concerned [11]. Nevertheless, this type of disturbance appears to be plausible, likely due to the possible involvement of multiple, remote associative bundles.

Different from the 11.5% seizure rate reported in the literature, our rate was higher (31%), likely due to our habit of also considering subclinical EEG alterations as seizures. In the literature, the issue of how to interpret EEG alteration in an asymptomatic patient is not well clarified. Fernandez-Torre et al. hypothesized that subclinical seizures were signs of glioma progression or recurrence [18]. Unluckily, the retrospective nature of our series does not permit us to divide clinical and subclinical seizures.

Postoperative KPS seems to be an important determinant of OS (three-times longer survival among patients with a KPS ≥ 70), given its impact on timing and quality of the adjuvant therapy. However, differently from single site HGG, MC HGG patients tend to lose functional independence (defined as a KPS ≥ 70) much earlier (after just three months).

A purely supratentorial extension was reported in the greatest part of patients. In the literature, there are few described cases of infratentorial MC or MF gliomas, and they appear burdened by a shorter OS. Our study confirmed Wang et al. in the fact that an infratentorial extension has a worse prognosis [14]. Parsa et al. stated that an ependymal involvement is associated with a more unfavorable outcome than unifocal disease [19]. In our clinical study, we had an ependymal involvement in seven cases. Although the small size does not make it possible to reach a conclusion, we can talk about a negative tendency with a mean OS of 6.8 months.

In general, we reported an OS of 8.7 months compared to 7.5 months from the literature review. There is no general consensus among studies available in the literature. In fact, Wang et al. obtained an OS of 9.7 months, while Di Russo et al. and Hassaneen et al. did not find differences with unifocal glioblastoma. Our results were probably determined by the strictness of inclusion criteria, with the exclusion of metacronous lesions.

The strong correlation between OS and postoperative KPS appears to agree with available data for unifocal HGG and, similarly to these latter, every effort meant to maintain a functional independence is absolutely necessary, with the aim of achieving the so-called maximal safe resection. Our study aimed to evaluate the role of surgery on the treatment of MC HGG, trying to understand if our clinical algorithm was useful for patients. Our data clearly showed that GTR or STR of the largest lesion confers an advantage compared to biopsy. Moreover, if we quantify this time gain, it appears that a “safe” surgery is probably the real factor that changes prognosis in patients with a MC HGG, just as for unifocal disease. Regarding patients undergoing a second resection, a clear advantage emerged from both clinical study and literature review. In particular, considering the latter, we found a quite similar survival to that reported by Hassaneen et al. in their study about multiple craniotomies [11]. This last finding could support the idea that, when feasible, removal of multiple lesions should be preferred.

Wang et al. conducted a study to establish outcome predictive factors in patients with a multicentric glioma. Univariate analysis revealed that an age ≤54 years was related to a better outcome. In our study, we choose a 70-year cut-off value because it is usually considered a limit for adjuvant treatment. In fact, older patents were excluded from the pivot study by Stupp et al. [20]. Therefore, our clinical series showed a clear survival advantage for patients younger than 70 years old, with an OS three times greater than for older patients (11.9 vs. 4 months); the same result emerged from the review and statistical analysis confirmed its significance. Comorbidity could represent a possible bias but, in our experience, only one patient died for complication not related to the tumor. Moreover, according to the literature review, only 25% of older patients were subjected to adjuvant radiotherapy. In their study about elderly patients with a unifocal HGG, Karsy et al. found that combined adjuvant therapy was associated with longer OS than chemotherapy alone [21]. On the contrary, we obtained no clear advantages for adjuvant therapy in this subclass of patients. In general, a significant correlation between OS and adjuvant combined treatment was demonstrated in our study; anyway, subsequent subanalysis showed the crucial role of chemotherapy in absence of a clear role of radiotherapy as contrast. This last finding collides with the study of Wang et al. that emphasized an important role of radiotherapy [14]. Therefore, we can assume that MC disease does not deviate from unifocal one so much, but which adjuvant treatment is superior is still object of study.

The 2016 WHO tumor classification attributed an important role to the molecular pattern of gliomas. In particular, it enhanced the role of IDH gene mutation. In fact, it seems related to a better response to Temozolamide and, as a consequence, to better prognosis. Both Carrillo et al. and Baldock et al. demonstrated a paradoxical correlation with a greater invasiveness on MRI sequences for IDH-mutated tumors [22,23]. Therefore, we could argue that MC and MF gliomas present mutation of IDH gene. On the contrary, Liu et al. conducted a study about genetic and epigenetic mutations of HGG and they concluded that prevalent pattern of MF and MC disease was mesenchymal [24]. Our study confirmed the above-mentioned findings with an almost total absence of IDH and ATRX mutations. Paradoxically, with the limits of sample size, the one patient with IDH mutation had the shortest OS. Karlowee et al. obtained an IDH, ATRX and p53 immunonegativity pattern, as a type 3 LGG or a “preglioblastoma”. Moreover, they registered EGFR amplification, a primary glioblastoma attitude, and we confirmed this finding [12]. Therefore, we could conclude that MC HGG does not seem arise from an LGG. In this context, the development of radiomics and the validation of results on molecular markers inferred by this method will likely have a major role in the diagnosis and management of lesions not amenable to total resection, and hopefully shed light on the true profile of multicentric versus multifocal high-grade gliomas [25].

## 5. Conclusions

MC HGG is a very rare condition with a higher prevalence among VI-VII life decade male patients and a short survival. Preoperative KPS does not influence OS, while the resection of principal, greatest lesion, seems to positively impact. A second resection should be performed whenever it is possible, with the aim to maintain a postoperative KPS ≥ 70. The latter was shown to represent a positive prognostic factor, as was adjuvant chemotherapy and/or combined treatment. Pathological examination revealed that MC HGG do not seem originate from an LGG, but deeper studies about molecular analysis are desirable in the near future.

## Figures and Tables

**Table 1:** *Patients preoperative clinical data*. M/S: motor and/or sensitive deficits; Seiz: seizures; Cogn/Aph: cognitive disorders and/or aphasia; H: headache; Localiz: localization; Epend: ependymal involvement.

#	Age	Sex	M/S	Seiz	Cogn/Aph	H	preKPS	Localiz	Epend
**1**	67	M		x			100	Supra	Yes
**2**	58	M	x		x		90	Supra	Yes
**3**	59	F			x		100	Supra	No
**4**	69	F		x			100	Supra	No
**5**	58	M	x				90	Supra	Yes
**6**	59	F	x		x		50	Supra	Yes
**7**	78	F		x	x		60	Supra	No
**8**	69	F		x	x		100	Supra	Yes
**9**	44	M				x	100	Supra	No
**10**	68	M	x				90	Supra/Infra	Yes
**11**	62	M				x	100	Supra	Yes
**12**	59	M		x			100	Supra	No
**13**	83	M			x		90	Supra	No
**14**	80	M	x		x		60	Supra	Yes
**15**	74	M	x				60	Supra	No
**16**	77	M	x		x		100	Supra/Infra	Yes

**Table 2:** *Postoperative data*. EOR: extent of resection; GTR: gross total resection; STR: subtotal resection; Che1: first-line adjuvant chemotherapy; RT: radiotherapy; Che2: second-line adjuvant chemotherapy; KPS3/6/12/18: 3, 6, 12, 18 months KPS; EOR2: second intervention extent of resection; OS: overall survival.

#	EOR	postKPS	Che1	RT	Che2	KPS3	KPS6	KPS12	KPS18	EOR2	OS
1	GTR	100	Yes	Yes		70	70	70	70		20
2	GTR	100	Yes	Yes		20	0	0			6
3	GTR	100	Yes	Yes		100	80	50			13
4	GTR	40	No	No		30	0	0			4
5	Biopsy	90	No	Yes		90	40	0			11
6	GTR	40	No	No		0	0	0			2
7	Biopsy	60	Yes	Yes		60	0	0			3
8	Biopsy	100				70	0	0			5
9	GTR	100	Yes	Yes		100	100	0		GTR	11
10	GTR	100	Yes	Yes		70	60	0			12
11	GTR	100	Yes	Yes		100	100	100	100		23
12	GTR	100	Yes	Yes		100	100	100	100	GTR	24
13	GTR	100	No	Yes		100	40	0			6
14	Biopsy	40	No	No		0	0	0			2
15	Biopsy	70	Yes	Yes	Yes	70	50	0			8
16	STR	50	No	No		0	0	0			1

**Table 3:** *Pathology*. GBM: glioblastoma multiforme; Olig III: anaplastic oligodendroglioma; AA: anaplastic astrocytoma; WT: wild type; TP53: percentuage of p53-mutated cells; EGFR: amplification of EGFR gene; ATRX: maintenance or deletion of ATRX gene.

#	Diagnosis	IDH1	ATRX	Ki67	TP53	EGFR	1p19q
1	GBM	WT	neg	10	5	No	No
2	GBM	WT	pos	60	10	Yes	No
3	GBM	WT	pos	20	5	Yes	No
4	GBM	WT	neg	60	60	Yes	No
5	GBM	WT	pos	35	20	Yes	No
6	GBM	WT	pos	70	20–30	Yes	No
7	GBM	WT	pos	40	30–35	Yes	No
8	GBM	WT	pos	50	70	Yes	No
9	GBM	WT	pos	10	0	Yes	No
10	GBM	WT	pos	50	20	Yes	No
11	GBM	WT	pos	10	5	Yes	No
12	GBM	WT	neg	40	30	Yes	No
13	Olig III	WT	pos	25	8	Yes	Yes
14	AA	WT	pos	10	0	Yes	No
15	GBM	WT	pos	35	50	Yes	No
16	GBM	mutated	pos	30	5	Yes	No

## Data Availability

Not applicable.
